# A Novel Cytoplasmic Male Sterility in *Brassica napus* (inap CMS) with Carpelloid Stamens via Protoplast Fusion with Chinese Woad

**DOI:** 10.3389/fpls.2017.00529

**Published:** 2017-04-06

**Authors:** Lei Kang, Pengfei Li, Aifan Wang, Xianhong Ge, Zaiyun Li

**Affiliations:** National Key Lab of Crop Genetic Improvement, National Center of Oil Crop Improvement (Wuhan), College of Plant Science and Technology, Huazhong Agricultural UniversityWuhan, China

**Keywords:** *Brassica napus*, *Isatis indigotica*, cytoplasmic male sterility, mitochondrial rearrangement, carpelloid stamen

## Abstract

A novel cytoplasmic male sterility (CMS) in *Brassica napus* (inap CMS) was selected from the somatic hybrid with *Isatis indigotica* (Chinese woad) by recurrent backcrossing. The male sterility was caused by the conversion of tetradynamous stamens into carpelloid structures with stigmatoid tissues at their tips and ovule-like tissues in the margins, and the two shorter stamens into filaments without anthers. The feminized development of the stamens resulted in the complete lack of pollen grains, which was stable in different years and environments. The pistils of inap CMS displayed normal morphology and good seed-set after pollinated by *B. napus*. Histological sections showed that the developmental alteration of the stamens initiated at the stage of stamen primordium differentiation. AFLP analysis of the nuclear genomic composition with 23 pairs of selective primers detected no woad DNA bands in inap CMS. Twenty out of 25 mitochondrial genes originated from *I. indigotica*, except for *cox2-2* which was the recombinant between *cox2* from woad and *cox2-2* from rapeseed. The novel *cox2-2* was transcribed in flower buds of inap CMS weakly and comparatively with the fertile *B. napus* addition line Me harboring one particular woad chromosome. The restorers of other autoplasmic and alloplasmic CMS systems in rapeseed failed to restore the fertility of inap CMS and the screening of *B. napus* wide resources found no fertility restoration variety, showing its distinct origin and the related mechanism of sterility. The reasons for the mitochondrial rearrangements and the breeding of the restorer for the novel CMS system were discussed.

## Introduction

Cytoplasmic male sterility (CMS) is a maternally inherited trait that fails to produce viable pollens, and has been reported in a large number of plant species. CMS is encoded in the mitochondrial genome and can arise spontaneously due to mutation in the genome (autoplasmy) or can be expressed following cytoplasmic substitutions due to nuclear-mitochondrial incompatibility (alloplasmy) (Prakash et al., 2009). A CMS fertility restoration system has been used as a suitable pollination control system to produce commercial hybrid seed for many crops, because it is easy to maintain.

Cytoplasmic male sterility phenotypes encompass a wide range of reproductive abnormalities, including degenerate anthers, aborted pollen, carpelloid and petaloid stamens (for review see Chase, 2007). Many CMS genes result from mtDNA rearrangements, as at least 10 essential mitochondrial genes are involved in the origination of CMS genes among 28 types of CMS from 13 crop species (Chen and Liu, 2014). Intriguingly, sequences of the known functional mitochondrial genes are not necessarily included in some CMS genes, for several rice CMS-associated ORFs consist of sequences of putative mitochondrial ORFs (Chen and Liu, 2014). Additionally, some CMS genes (such as *orf125* in radish CMS-Kos and its variant *orf138* in *Brassica* CMS-Ogu) are non-chimeric genes with the sequences from single source (Bonhomme et al., 1992; Iwabuchi et al., 1999). CMS genes have been shown to cause mitochondrial dysfunction by disrupting the mitochondrial membranes, reducing the mitochondrial ATP level and increasing the reactive oxygen species content (Hanson and Bentolila, 2004; Chen and Liu, 2014; Horn et al., 2014). Up to now, nine *Rf* (*restorer of fertility*) genes have been isolated in seven plant species, such as *Rfo* (*Rfk1*) in radish and *Brassica* (Chen and Liu, 2014). Most of the identified *Rf* genes encode PPR (pentatricopeptide repeat) proteins, but *Rf* genes are also highly multifarious and the respective restoration of fertility in CMS/*Rf* systems may be realized by various mechanisms at genomic, post-transcriptional, translational, or post-translational, and metabolic levels (Chen and Liu, 2014).

Stable CMS lines of crops could be produced by introducing the alien cytoplasm from the relatives through interspecific-/intergeneric hybridizations, or by mediating the mitochondrial rearrangement via somatic fusions (Prakash et al., 2009). For *Brassica* crops, since a sterility inducing cytoplasm identified in a wild population of *Raphanus sativus* (Ogura CMS) was introduced into *B. napus* and *B. oleracea* (Bannerot et al., 1974), a spectrum of alloplasmic CMS lines of diverse origins have been obtained by combining the cytoplasm of *Brassica* coenospecies with crop nuclei, particularly in *B. juncea* (see reviews by Delourme and Budar, 1999; Budar et al., 2004; Prakash et al., 2009; Yamagishi and Bhat, 2014). More recently, a novel CMS system in *B. juncea* incorporating the cytoplasm of *B. fruticulosa* was developed (Atri et al., 2016).

Somatic hybridization via protoplast fusion is a possible alternative for gene transfer from wild relatives to crops, by combining both nuclear and cytoplasmic genomes of two distantly related species, genera or even tribes. In somatic hybrids from protoplast fusion, the chloroplasts are usually inherited from one of the parents, whereas the mitochondrial DNA (mtDNA) is rearranged and may include DNA from both parents (Prakash et al., 1999). Intergenomic mitochondrial recombination with high frequency was found to occur in various somatic hybrids from the combinations of different species within *Brassicaceae* (Landgren and Glimelius, 1994; Dieterich et al., 2003; Leino et al., 2003; Oshima et al., 2010; Liu et al., 2015), leading to novel CMS genes. In the *B. napus* CMS Tournefortii-Stiewe produced by protoplast fusion with *B. tournefortii* (Dieterich et al., 2003), the mitochondrial rearrangement at upstream of the gene *atp9* generated a chimeric *orf193* that was co-transcribed with *atp9*. But another *B. napus* alloplasmic CMS Tournefortii by sexual hybridization with the same species had a chimeric *orf263* at the vicinity of the *atp6* (Landgren et al., 1996). Furthermore, recombinant mitochondrial genomes were also generated when protoplasts of fertile and sterile cytoplasm were fused (Sakai and Imamura, 1990; Wang et al., 1995; Meur et al., 2006).

From the mitochondrial genomes of nap, pol, ogu, and hau CMS sequenced (Handa, 2003; Chen et al., 2011; Tanaka et al., 2012; Heng et al., 2014), comparative analysis revealed that the CMS associated genes were localized close to the edge of syntenic sequence blocks, together with short repeats and overlapped repeats. These repeats were responsible for reorganization of mitochondrial genomes via homologous recombination. Overlapping homologous sequences from the fusion parents were produced when the parental mitochondria fused and the recombination occurred. In the cybrid derived from the somatic hybrid between *Nicotiana tabacum* and *Hyoscyamus niger* after being backcrossed with *N. tabacum*, the mitochondrial genome was highly recombinant with at least 35 intergenomic rearrangement events detected between two parents (Sanchez-Puerta et al., 2015). Recombination occurred via homologous mechanisms involving the double-strand break repair and/or break-induced replication pathways.

Currently, the autoplasmic pol CMS system from natural mutations in *B. napus* is still predominant for rapeseed hybrid production in China, but the alloplasmic Ogura CMS-fertility restoration system is widely used in Europe and Canada. Although several CMS systems have been reported, they are not used commercially due to undesirable CMS phenotype and lack of fertility restoration genes. In our previous study, the intertribal somatic hybrids between *B. napus* and *Isatis indigotica* Fort. (Chinese woad) of the *Isatideae* tribe within the *Brassicaceae* family were obtained and backcrossed successively to *B. napus* (Du et al., 2009; Kang et al., 2014), resulting in the development of one novel *B. napus* CMS line with carpelloid stamens (named as inap CMS). Herein, the histological and genetical characterizations of inap CMS were described, together with the mitochondrial rearrangements.

## Materials and Methods

### Plant Materials

**FIGURE 1:**
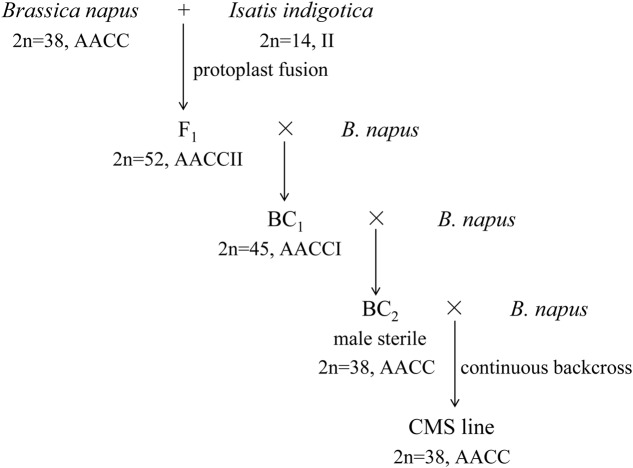
**The breeding procedure of inap cytoplasmic male sterility (CMS) line**.

The intertribal somatic hybrid (As1, 2*n* = 52, AACCII) between *B. napus* L. cv. Huashuang 3 (2*n* = 38, AACC) and *I. indigotica* Fort. (Chinese woad, 2*n* = 14, II) was previously produced by protoplast fusion (Du et al., 2009). The backcrossed progenies with the same chromosome number and complement as *B. napus* were male sterile due to the development of carpelloid stamens (Kang et al., 2014). The phenotype of the carpelloid stamens was stably maintained after backcrosses with Huashuang 3 (**Figure 1**), which showed the characteristic of the cytoplasmic inheritance. Then, the novel CMS line of *B. napus* was developed and named as inap CMS. Its stability of male sterility was observed for 3 years in two locations and two seasons: Qinghai University experimental field in summer (Xining, altitude 2261 m, 101°77′E, 36°62′N, average temperature 19°C) and Huazhong Agricultural University in spring.

### Nuclear and Organelle DNA Analysis

For nuclear analysis, total DNA was extracted and purified from young leaves of the parents and CMS line according to the method by Dellaporta et al. (1983). AFLP analysis was carried out according to the procedures of Vos et al. (1995) with some modifications. Genomic DNA (75 ng) of each sample was digested by using the restriction endonucleases *EcoR* I and *Mse* I for 6 h at 37°C and denaturalized for 1 h at 65°C. Then two adapters were ligated to the sticky ends of the digested DNA at 22°C overnight, and the resulting ligation products were amplified by PCR with primers matching the adapters. After being diluted to 15 times, resultant PCR products were amplified using 23 pairs of randomly selected primers. Finally, the PCR products were separated on 6% polyacrylamide gels and DNA bands were visualized by silver staining.

For mtDNA analysis, 24 mitochondrial genes were examined in parents and CMS line (Supplementary Table S1). A total of 20 μl reaction contained 4 μl 5Phusion HF Buffer, 0.4 μl 10 mM dNTPs, 0.5 μl forward and reverse primer (10 μM), 0.6 μl 100% DMSO, 1 μl template DNA (50 ng/μl) and 0.2 μl Phusion Hot Start II High-Fidelity DNA Polymerase (2 U/μl). PCR amplification was carried out with an initial denaturation step at 98°C for 30 s followed by 35 cycles of 94°C for 5 s, 65°C for 20 s (optimal annealing temperature was determined by Tm calculator on website: www.thermoscientific.com/pcrwebtools), 72°C for 45 s, and a final10-min extension at 72°C. PCR products were separated on 0.8% agarose gels and purified using the TIANgel Midi Purification Kit (TIANGEN BIOTECH (BEIJING) CO., LTD., Beijing, China). The 3′-A overhang of blunt-end DNA fragments was added using DNA A-Tailing Kit (TaKaRa BIO INC, Dalian, China). Then the products were cloned into the pMD18-T vector and sequenced. The primers for amplifying complete sequence of *cox2-1* and *cox2-2* were designed based on the rapeseed mtDNA sequence (accession number: AP006444, **Table 1**).

**Table 1:** The primer sequences for amplifying the complete sequence of *cox2-1* and *cox2-2.*

Primer names	Primer sequences (5′–3′)
F1	GAGCGGAGCAGTCAATGAAG
F2	TAGGAGTGTGAGCAGTACGAG
R1	CCCTCCCTCACCTTACTCTTC
R2	TGCACCATATTTTGATCTGCC
R3	GGTCAGCTTTCTTTGGCATCT


To distinguish inap CMS from Ogura and pol CMS, the primers of *orf138* (GenBank: AB055443) and *orf224* (GenBank: EU254235) were designed (Supplementary Table S1). Reactions (10 μl) contained 1 × *Taq* buffer, 2 mM MgCl_2_, 5 mM dNTPs, 5 μM forward and reverse primer, 1 U *Taq* DNA polymerase and 50 ng total DNA. DNA fragments were amplified after a 5-min denaturation at 94 for 30 cycles (94°C for 45 s, 57°C for 30 s, 72°C for 90 s), and a 10-min extension step at 72°C.

### Reverse Transcription-PCR

Total RNA was extracted from flower buds of inap CMS line, *B. napus* cv. Huashuang 3, *I. indigotica* and the male fertile *B. napus* monosomic addition line Me (Kang et al., 2014) using the Eastep^®^ Super Total RNA Extraction Kit (Shanghai Promega Biological Products, Ltd, Shanghai, China). cDNA was synthesized from total RNA using the RevertAid First Strand cDNA Synthesis Kit (Thermo Fisher Scientific Inc., America). RT-PCR amplification was carried out with an initial denaturation step at 94°C for 5 min followed by 35 cycles of 94°C for 30 s, 57°C for 30 s, and 72°C for 90 s, and a final 10-min extension at 72°C.

### Histological Studies

The inflorescences of *B. napus* and CMS line were first fixed by 50% FAA solution and stained with Ehrlich’s haematoxylin solution. After rinsed with distilled water, the samples were dehydrated with a graded ethanol series (15, 30, 50, 70, 85, 95, and 100%) for 4 h in each. Then samples were gradually cleared in 20, 40, 60, 80, and 100% chloroform for 2 h, and added paraffin fragments into 100% chloroform twice and kept in an incubator at 37°C for 2 days. The samples were embedded in Paraffin with ceresin (Sinopharm Chemical Reagent Co., Ltd, Shanghai, China). The tissues were sectioned into 8 μm sections, floated on drops of distilled water and dried overnight onto Poly-L-Lysine-coated slides at 37°C. After deparaffinized with xylene, the tissues sections were mounted by neutral balsam. All micrographs were taken with a Nikon Digital DS-Ri1.

For scanning electron microscopy, the young flower buds were fixed with 2.5% glutaraldehyde in 0.05 M phosphate buffer (pH 6.8) at room temperature for 2 h, then post-fixed in 1% osmium tetroxide in the same buffer at 4°C for 1 h. The samples were dehydrated with a graded ethanol series, then critical point dried using liquid CO_2_. After mounting, the samples were shadowed with gold and observed with a JSM-6390LV scanning microscope (NTC, Japan).

### Restorer Search for inap CMS

The restorer lines of different CMS systems were used to test their ability to restore the fertility of inap CMS (**Table 2**). The seeds of Ogura and pol CMS lines and restorer lines were kindly provided by Prof. Jiangsheng Wu from our University. *B. napus* cv. Yudal was reported to restore Tournefortii-Stiewe CMS with recombinant mtDNA of both *B. tournefortii* and *B. napus* (Dieterich et al., 2003). The disomic rape-radish addition line (DAL-f) (2*n* = 40, AACC+1II_R_) was selected, for it acted as the restorer for a novel CMS of *B. napus* with *Raphanus* cytoplasm and also carpel-like stamens (Budahn et al., 2008). However, the addition with the chromosome F did not restore Ogura CMS of *B. napus*. One *B. napus* monosomic addition line Me carrying one chromosome of Chinese woad and the same cytoplasm of inap CMS was used, as it produced anthers with viable pollen grains (Kang et al., 2014). After inap CMS line was pollinated by these different lines by hand, the seeds were harvested and F_1_ plants were assessed for the degree of male sterility.

**Table 2:** The restorers of other cytoplasmic male sterility (CMS) lines and the male fertility of their progenies with inap CMS.

Restorer lines	CMS restored	Restoration for inap CMS
6010	Ogura CMS	S
6012	Ogura CMS	S
8075	pol CMS	S
Westar	nap CMS	S
Yudal	Tournefortii-Stiewe CMS	S
DAL-f	*Raphanus* cytoplasm CMS	S
Me	inap CMS	F


*S, sterile; F, fertile.*

## Results

### Phenotype of inap CMS in *B. napus*

**FIGURE 2:**
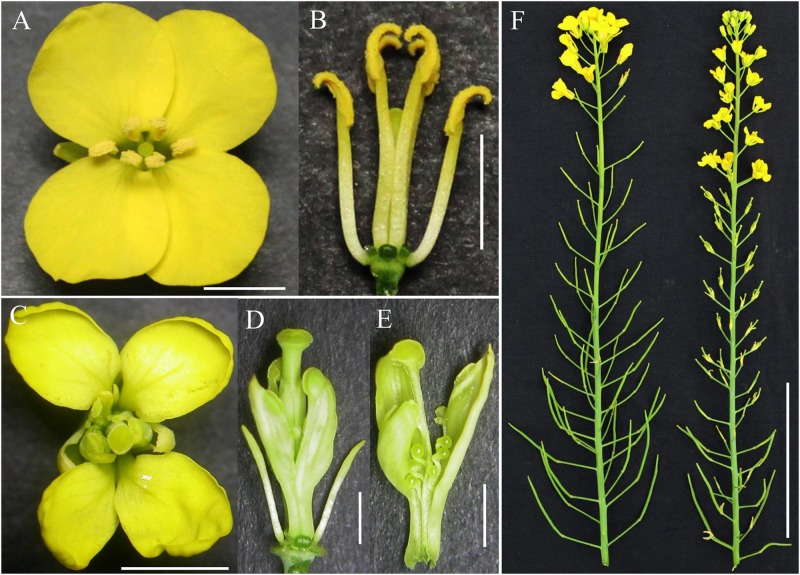
**Flower phenotype of inap CMS and *Brassica napus* donor.** **(A,B)** The opened flower and the one with sepals and petals removed of Huashuang 3. **(C,D)** The whole flower and that with sepals and petals removed of inap CMS. **(E)** The carpelloid stamen with stigmatoid structures at its tips and ovules in the margins. **(F)** The inflorescences of Huashang 3 (left) and the CMS (right). Bars: **(A–C)** 5 mm; **(D,E)** 2 mm; **(F)** 10 cm.

The inap CMS of *B. napus* was selected from the somatic hybrids with Chinese woad by successive backcrossing to recover the *B. napus* chromosome complement (**Figure 1**). The male sterility was attributed to the developmental conversion of its tetradynamous stamens into the carpelloid structures with stigmatoid tissues at the tips and ovule-like tissues in the margins, and two shorter stamens into filaments (**Figures 2A–E**). The two carpelloid structures at the same side fused together in some flowers, and still remained after the flower faded and as the pods developed (**Figure 2F**). The size and developmental complex of carpelloid structures diminished after successive backcrosses. The petals and sepals of its flowers were significantly smaller than the donor *B. napus* cv. Huashuang 3 (**Table 3**), and the number of nectaries reduced to 2.4 from 4 of Huashuang 3 due to the fused carpelloid stamens. Although the pistil length of the flowers was close to Huashuang 3, the seeds per pod (16.1) of inap CMS after pollination by Huashuang 3 were fewer than those in Huashuang 3 (23.5) by selfing (**Table 3**), likely some ovules were inviable (24.9/30.9). The negative effect of the cytoplasm from inap CMS on the defect of female development might cause the yield penalty at certain degrees, and optimal combinations needed to be screened for its correction. The male sterility phenotype was expressed consistently during recurrent backcrosses, and in Wuhan and Xining for 3 years, indicating its genetic and environmental stability. Then the male sterility was also easily maintained by Huashuang 3 and all other *B. napus* genotypes used (see below), as inap CMS had good seed-sets and still produced the male sterile progenies after pollinated by them.

**Table 3:** Flower traits and fertility of inap CMS and *B. napus* (H3).

Line	Sepal length (cm)	Sepal width (cm)	Petal length (cm)	Petal width (cm)	Pistil length (cm)	No. of nectaries	Ovules/ovary	Seeds/silique	1000 seeds weight (g)
H3	0.83 ± 0.04	0.29 ± 0.03	1.48 ± 0.09	0.85 ± 0.06	0.81 ± 0.06	4.00 ± 0.00	30.60 ± 2.73	23.50 ± 1.20	4.25 ± 0.04
CMS	0.66 ± 0.03^∗∗^	0.21 ± 0.02^∗∗^	1.10 ± 0.06^∗∗^	0.53 ± 0.05^∗∗^	0.84 ± 0.08	2.40 ± 0.80^∗∗^	24.90 ± 1.37^∗∗^	16.10 ± 2.34^∗∗^	4.43 ± 0.06^∗^


*^∗^Means *p* < 0.05 by *t*-test. ^∗∗^Means *p* < 0.01 by *t*-test.*

The plants of inap CMS grew more slowly and flowered later almost 2 weeks than Huashuang 3 in Wuhan, but they show no leaf chlorosis, likely for they maintained the chloroplast genetic component from Huashuang 3 (Du et al., 2009; Kang et al., 2014). But death of flower buds occurred at the initial stage of flowering, and later the flower buds developed and opened normally.

### Stamen Development of inap CMS

**FIGURE 3:**
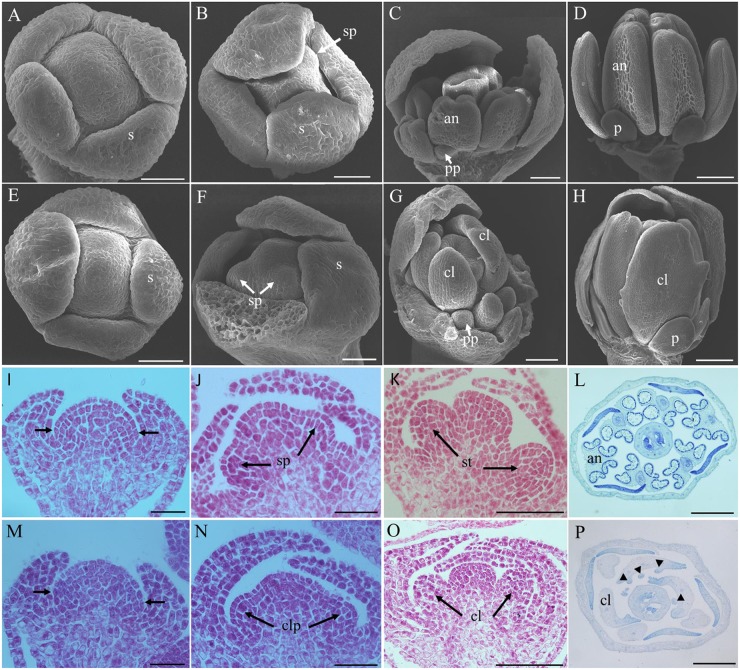
**Comparative histology of flower development in *B. napus* and inap CMS line.** **(A,E)** Flower buds at stage 4 of *B. napus* and inap CMS. **(B,F)** The stamen primordia are visible (arrows) at stage 5. **(C,G)** At stage 7, the anthers are visible in *B. napus*, while the stamen are converted to the carpelloid structure in inap CMS line. **(D,H)** Flower buds of *B. napus* and inap CMS at stage 9. **(I,M)** Flower buds at stage 4. Arrows indicate the sites where the stamen primordium and petal primordium will arise. **(J,N)** Flower buds at stage 5. **(K,O)** Flower buds at stage 6. **(L,P)** Cross sections of flower buds of *B. napus* and inap CMS. Triangles indicate ovule-like structures on the carpelloid organs in inap CMS. an, anther; cl, carpelloid structure; clp, carpelloid primordium; pp, petal primordium; p, petal; s, sepal; sp, stamen primordium; st, stamen. Bars: **(A,B,E,F,K,O)** 50 μm; **(C,G,L,P)** 100 μm; **(D,H)** 200 μm; **(I,J,M,N)** 20 μm.

The stages of floral development in *Arabidopsis thaliana* were defined by Smyth et al. (1990) using scanning electron microscopy. With this standard, the early flower developmental processes of inap CMS line and its maintainer line Huanshuang 3 were observed and compared. They showed no differences until the stamen primordium appeared (**Figures 3A,B,E,F**). Subsequently, the stamen primordium developed into stamen in the maintainer (**Figures 3C,D**), while the stamen converted into the carpelloid structure in the CMS (**Figures 3G,H**). The ovule-like structure appeared on the carpelloid tissues in the CMS line at the stage 9 (data not shown). By histological studies, disordered cell division occurred at the sites of the stamen primordium in the flower buds at stage 4, and petal primordium would arise (**Figures 3I–P**). The stamen primordium developed into the carpelloid structure as a result of disorganized cell proliferation. So the male sterility of inap CMS initiated at the stage of stamen primordium differentiation.

### Nuclear and Cytoplasmic Composition of inap CMS

With randomly selected 23 pairs of AFLP selective primers, no *I. indigotica* genomic bands were found in inap CMS line, showing no woad chromosomal segments introgressed after successive backcrosses with *B. napus*.

**FIGURE 4:**
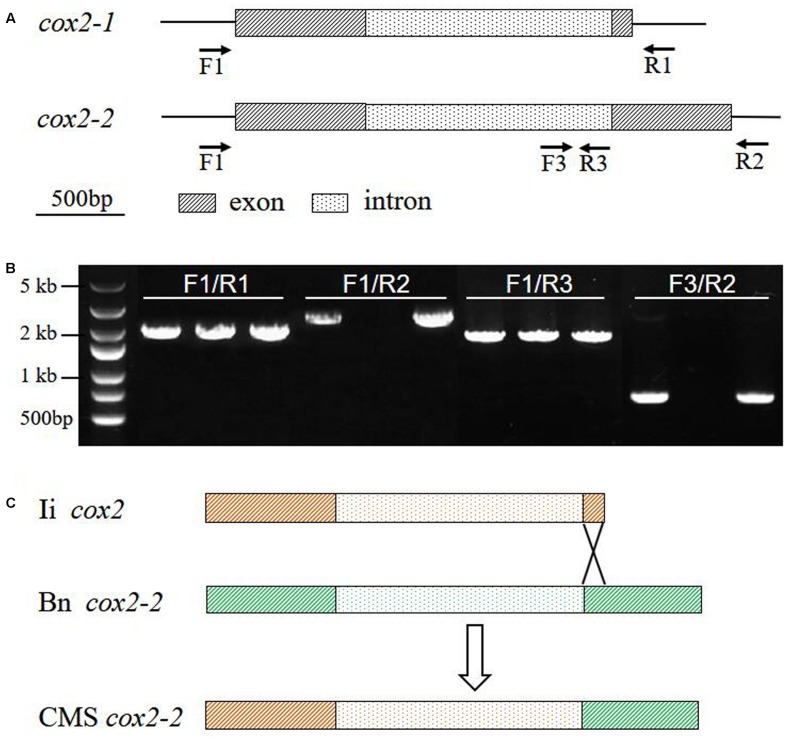
**The origin of *cox2-2* in inap CMS.** **(A)** The binding sites of primers used for specific amplification of mtDNA fragments. **(B)** PCR amplification products of mtDNA fragments from *B. napus*, *I. indigotica* and inap CMS. **(C)** Gene structures, showing that the gene *cox2-2* of inap CMS was the recombinant from *cox2* of *I. indigotica* and *cox2-2* of *B. napus* via homologous recombination. Bn, *B. napus*; Ii, *I. indigotica*.

It has been shown that the somatic hybrid contained the recombinant mitochondrial genome from *B. napus* and *I. indigotica* (Kang et al., 2014). As most of mitochondrial genes had no fragment length differences between parents and the hybrid from PCR amplification, 25 mitochondrial genes in the CMS line were sequenced and found that 20 of them originated from *I. indigotica* (**Table 4**). Only the genes *atp9*, *cox3*, *rps7*, and *rrn26* showed no sequence differences between parents and CMS line. Several SNPs were identified at the first exon and the intron of *cox2-2* between CMS and *B. napus*, while one same band was detected by PCR amplification using its primers. Two copies of *cox2* genes were reported to exist in *Brassica* species, except for *B. nigra* and *B. carinata* (Handa, 2003; Yamagishi et al., 2014), which showed a consistent sequence of the first exon and intron and diverged from each other at the second exon. The *cox2-1* was homologous to the mitochondrial *cox2* genes of other plants, but *cox2-2* had an extension with no homology to any other sequences. To obtain the full sequence of *cox2-1* and *cox2-2* of inap CMS and parents, the primers were redesigned (**Table 1** and **Figure 4A**). Only one copy of the *cox2* (GenBank: KY656165) was obtained from *I. indigotica*, having 99.77% sequence identity with *cox2-1* of *B. napus* (**Figure 4B** and Supplementary Figure S1). Multiple sequence alignment indicated that the first exon and intron of the *cox2-2* of the CMS was from the *cox2* of *I. indigotica*, but the second exon was derived from the *cox2-2* of *B. napus* (**Figure 4** and Supplementary Figure S1). RT-PCR analysis showed that this *cox2-2* was transcribed in inap CMS flower buds, but the expression level was very low (Supplementary Figure S2). To examine the effect of nuclear restorer gene on the expression of the *cox2-2*, the expression level was compared between the fertile *B. napus* addition line Me and inap CMS, but showed no significant difference (Supplementary Figure S2).

**Table 4:** The origin of mitochondrial genes and their differences in the inap CMS.

Gene	*B. napus*	*I. indigotica*	inap CMS	Difference
*atp1*	B	I	I	SNP
*atp4*	B	I	I	SNP
atp6	B	I	I	SNP
*atp8*	B	I	I	SNP
*atp9*	S	S	S	-
*ccmB*	B	I	I	SNP
*ccmC*	B	I	I	SNP
*ccmFC*	B	I	I	SNP
*cob*	B	I	I	SNP
*cox1*	B	I	I	SNP
*cox2-1*	B	I	I	SNP
*cox2-2*	B	-	R	SNP
*cox3*	S	S	S	-
*matR*	B	I	I	SNP
*nad3*	B	I	I	SNP
*nad6*	B	I	I	SNP
*nad9*	B	I	I	SNP
*rpl5*	B	I	I	SNP
*rpl16*	B	I	I	SNP
*rps3*	B	I	I	Indel, SNP
*rps4*	B	I	I	SNP
*rps7*	S	S	S	-
*rps12*	B	I	I	SNP
*rrn18*	B	I	I	Indel, SNP
*rrn26*	S	S	S	-


*B, *B. napus*; I, *I. indigotica*; R, recombination gene; S, same nucleotide sequence.*

To determine whether inap CMS was different from Ogura and pol CMS in sterility genes, the primers of their respective CMS-associated genes *orf138* and *orf224* were used for PCR amplification, but did not produce the products, showing that inap CMS did not share these genes (Supplementary Figure S3).

### Different Fertility Restoration Relationship of inap CMS from Other CMS Systems

As shown in **Table 2**, all the F_1_ hybrids between inap CMS and these restorers of various CMS systems were male sterile and still produced the carpelloid stamens, except for those with the *B. napus* addition line harboring the woad chromosome E. Although the two alloplamic Ogura CMS lines had the similar phenotype of inap CMS, their restorers with the introgression of restoration gene from one specific radish chromosome F failed to restore our inap CMS. Furthermore, the hybrids between inap CMS and the disomic rape-radish chromosome addition line f (DAL-f) (2*n* = 39, AACC+1R) with the radish chromosome carrying the restoration gene still displayed male sterility. To identify restorers of inap CMS in *B. napus*, 112 cultivars of wide origins were used as pollinators (data not shown) and the pods with good seed-sets were produced, but none was found to function as the restorer.

## Discussion

The novel inap CMS in *B. napus* selected from the intertribal somatic hybrids contained the recombinant mtDNA of both parents but biased to woad, and had the stamens converted into carpelloid structures. The sterile phenotype was stable during years and under different ecological environment. The developmental conversion of stamens occurred at the stage of stamen primordium differentiation. It was different from other CMS lines in phenotype, CMS associated genes, and restorer and maintainer relationships.

Homeotic conversion of stamens into carpelloid structures were described in several CMS lines of wheat (Ogihara et al., 1997), tobacco (Farbos et al., 2001), carrot (Linke et al., 2003), rapeseed (Leino et al., 2003), cauliflower (Kamiñski et al., 2012), and broccoli (Shu et al., 2015). Stamens of the CMS lines were replaced by carpelloid organs, thus resembled the B-class genes *APETALA3* (*AP3*) and *PISTILLATA* (*PI*) mutants. In *B. napus* CMS line with the rearranged *Arabidopsis* mitochondria, downregulation of *AP3* in the stamen primordia and subsequent repression of *PI* resulted in the conversion of stamens into carpelloid organ (Carlsson et al., 2007a). Similarly, the B-class genes were transcriptionally downregulated in carpelloid CMS flowers of tobacco, carrot and wheat (Farbos et al., 2001; Linke et al., 2003; Hama et al., 2004). The mitochondrial background had a distinct influence on nuclear gene expression (Carlsson et al., 2007b). This indicated that CMS-associated mitochondria genes regulated the expression of these nuclear MADS-box genes by mitochondrial retrograde signaling (MRS). *Wheat Calmodulin-Binding Protein 1* (*WCBP1*) significantly upregulated in young spikes of the pistillody line (Yamamoto et al., 2013), indicating that CMS- associated genes played a role in development of pistil-like stamens by MRS involved Ca^2+^ signaling pathway in wheat CMS.

The most mitochondrial genes of inap CMS were found to originate from *I. indigotica* (**Table 4**), which was likely caused by the asymmetric fusion to produce the hybrids (Du et al., 2009). The protoplasts of *B. napus* were treated by 3 mM iodoacetate (IOA), and those of *I. indigotica* were irradiated with UV (Du et al., 2009). IOA could inactivate the glyceraldehyde-3-phosphate dehydrogenase, and resulted in the prevention of the glycolytic pathway (Zhao et al., 2008). Only when *B. napus* protoplasts for fusion contained the normal cytoplasm, the fused cells could divide, formed calli and regenerated plants. Thus, the mitochondria from *I. indigotica* were retained, and those from *B. napus* eliminated but several segments were introgressed into the mitochondria of the hybrid by recombination (**Table 4**). Such situation was also reported in other asymmetric somatic hybrids (Morgan and Maliga, 1987; Akagi et al., 1995). MtDNA sequencing of an Ogura-CMS cybrid derived from somatic fusions between *B. napus* and sterile radish showed that the mtDNA component mainly inherited from radish (Wang et al., 2012). The *B. napus* origin of the chloroplast DNA of inap CMS was in accord with the conclusion that the chloroplasts were generally from the iodoacetate-treated parent (Morgan and Maliga, 1987; Leino et al., 2003). This might be due to the better compatibility of recipient chloroplasts with the recipient nucleus, or the loss of the irradiated chloroplasts.

Two copies of *cox2* gene in *Brassica* species (Handa, 2003; Yamagishi et al., 2014) arose from duplication, for it was involved in a large repeat sequence. Via this large repeat, the *Brassica* mitochondrial genome could be recombined into two subgenomic circles. *I. indigotica* contained only one copy of this gene. Of two copies of *cox2* gene in inap CMS, *cox2-1* was from *I. indigotica*, but novel *cox2-2* was the recombinant between *cox2* of woad and *cox2-2* of rapeseed (**Figure 4** and Supplementary Figure S1). Rearrangements in the *cox2* region were also reported in somatic hybrids between *B. napus* and *A. thaliana* (Landgren and Glimelius, 1994; Leino et al., 2003), *B. napus* and *R. sativus* Kosena CMS (Sakai and Imamura, 1990). CMS-associated genes identified in many CMS systems (Wang et al., 1995; Chen et al., 2011; Tanaka et al., 2012; Heng et al., 2014) were located in flanking sequence of the known mitochondrial genes, and co-transcribed with it. Protoplast fusion offered an opportunity for mitochondrial genome recombination, leading to appearance of novel CMS genes. Three *atp9* genes were present in the alloplasmic Tournefortii-Stiewe CMS of *B. napus*, which was generated by protoplast fusion with *B. tournefortii* (Dieterich et al., 2003). MtDNA rearrangements happened in the upstream of one of these genes and generated a chimeric gene *orf193* that was co-transcribed with *atp9*. An alloplasmic *B. juncea* CMS line derived from somatic hybridization with *Diplotaxis catholica* had two copies of *coxI* gene, and the recombinant *coxI-2* gene was suggested to cause CMS (Pathania et al., 2007). For the novel *cox2-2* gene was expressed in the inap CMS flowers weakly and insignificantly from restorer plant, it was likely not responsible for male sterility.

The identification of the restorer and maintainer relationship is one of the most classical methods to distinguish different types of sterile cytoplasm (Wan et al., 2008). The test results showed that the fertility of inap CMS failed to be restored by restorer lines from Ogura, pol, nap and Tournefortii-Stiewe CMS systems. Budahn et al. (2008) found that rapeseed plants with the alien radish cytoplasm showed pistilloid stamens with ovules and stigmatoid tips, and the radish chromosome F could eliminate the disadvantageous effect of alien cytoplasm in *B. napus*. Additionally, the chromosome F did not restore Ogura CMS. In spite of the similar phenotype with inap CMS, the radish chromosome F was unable to restore its fertility, indicating that these two CMS lines had different CMS-associated genes and mechanisms of male sterility. No restorers were screened among 112 *B. napus* cultivars. This further testified that the fertility restoration gene(s) of alloplasmic CMS lines generally existed in nuclear genome of cytoplasm donor species. The restoration gene of a *B. napus* CMS line with rearranged *A. thaliana* mtDNA was located on chromosome III of *A. thaliana* (Leino et al., 2004). The restorer line for *Nsa* CMS of *B. napus* developed by the somatic hybridization with *Sinapis arvensis* was one disomic addition line with the restoration gene on the alien chromosome (Wei et al., 2010). Similarly, the restoration gene(s) for our inap CMS also existed on one specific woad chromosome, because only the monosomic alien addition line of *B. napus* with woad chromosome e among the complete set of such lines and also the same cytoplasm as inap CMS produced the normal flowers with fertile pollen grains (Kang et al., 2014). To breed the restorer line for inap CMS, the chromosomal segment carrying the fertility restoration gene(s) should be introgressed into one of rapeseed chromosome and the lines (2*n* = 38) with high restoration capability selected, as excellently exemplified by the long-term development of the radish introgression carrying the Rfo restorer gene for the Ogu-INRA CMS in *B. napus* (Delourme et al., 1991, 1998, 1999; Primard-Brisset et al., 2005). The identification of some progeny plants (2*n* = 38) with the restored flower development and pollen fertility from the addition line gave the expectation that the new CMS and fertility restoration system should be developed for rapeseed hybrid production in near future.

## Author Contributions

ZL and LK conceived and designed the experiments, analyzed the data and wrote the paper. LK performed the experiments. PL participated in mtDNA analysis. AW performed the histological section experiments. XG provided technical expertise for molecular analysis and scientific discussions.

## Conflict of Interest Statement

The authors declare that the research was conducted in the absence of any commercial or financial relationships that could be construed as a potential conflict of interest.
