# Supplementary material for: A Novel Cytoplasmic Male Sterility in Brassica napus (inap CMS) with Carpelloid Stamens via Protoplast Fusion with Chinese Woad
Source: Front Plant Sci. 2017 Apr 6;8:529. doi: 10.3389/fpls.2017.00529 (PMC5382163; doi:10.3389/fpls.2017.00529)
Supplement: Supplementary file 1 [file Table_1.DOCX]

Supplementary Material

A novel cytoplasmic male sterility in *Brassica napus* (inap CMS) with carpelloid stamens caused by mitochondrial DNA rearrangement via protoplast fusion with Chinese woad

Lei Kang *, Pengfei Li, Aifan Wang, Xianhong Ge, Zaiyun Li

*** Correspondence:** Zaiyun Li, [lizaiyun@mail.hzau.edu.cn](mailto:lizaiyun@mail.hzau.edu.cn)

# Supplementary Table

**Supplemental Table 1.** The primers of mitochondrial genes used for PCR in this study

| gene | Forward primer (5’-3’) | Reverse primer (5’-3’) |
| --- | --- | --- |
| *atp1*^a^ | AGGGCTTGTAGTAATTCAGGT | GTCGGGATTGTTGTCTTTGGT |
| *atp4*^a^ | TCTTAAGACCACCAAGCTCTC | AGTTCGACGAATATGGATGCT |
| *atp6*^a^ | GCTACACCTAATTCCAGACCG | TTGTTCATGCTGCTAACTCTG |
| *atp8* | TTCCTACAAGTGATCCACCT | CACAATTCTTCTGGTTATGC |
| *atp9*^a^ | GGAGCTGCTACAATTGCTTC | ACAATGCAATAGCTTCGGTT |
| *ccmB*^a^ | CATAAACTGATCTTCCCCTCC | AGACCGAAATTGGAAAAAGAG |
| *ccmC*^a^ | CGCAAATTCTCATTGGGTCTT | TGGATGCAAGCGAGTGAACTA |
| *ccmFC*^a^ | AGTCGCCCTATTCTATTACCAG | CTGACGTAACAAACTAGGCAAG |
| *cob*^a^ | GTAGATTATCCAACCCCGAGC | CTTCCAACTCGTCCCAGAATG |
| *cox1*^a^ | TTTCGTCTCCTTGATAGCTGG | CCTATTTGTGTGGTCCGTTC |
| *cox2-2*^a^ | ATTTCTCCTTGTGATGCAGCG | GATCCGCTCCCATTTTTGACA |
| *cox3*^a^ | TCGTACAATTAGGACCTCGAT | TCCCCACCAATAGATAGAGACA |
| *matR*^a^ | AATCCATTTACGATCCCGAGT | TTTTGCTCATCCCCTTCGTCA |
| *nad3*^a^ | TTCTTTGATCCTACTCGGTGT | GAAGCACCCCTTTTCCATTCA |
| *nad6*^a^ | ACTTTCTGTTTTGTCGAGCCC | AGTAGATCGTGAGTGGGTCAG |
| *nad9*^a^ | TCCCAAGGACTAGCAAAATCG | ATGGAAAGATCGGAACATGGG |
| *rpl5*^a^ | AGTTCCTGGATCGTGTGAAAT | GTTGGCCGAAGTGACAATAGT |
| *rpl16*^a^ | GTTACGGAATCTCAGGTGTCA | ATGGTTTATGAGCCGCTAATG |
| *rps3*^a^ | ATTCAAGTCGGTTCAGTGAGT | TCCACACTTTGACACCTGAGA |
| *rps4* | CGAAGAGGAAGGTTTGGATCT | TCCGAAGATTGAGGAACAGGA |
| *rps7*^a^ | CATCTGAAATGCGCGAAACTT | CTTGGATGGTGAGCAAAAACT |
| *rps12*^a^ | CCGTACTCGAGCTTTGGATAA | TGCACCATATTTTGATCTGCC |
| *rrn18* | ACGGCTACCTTGTTACGAC | CAAGAGGTGGGAAGAGTTGTG |
| *rrn26* | CTCCGAATACAGGCCGTTCT | TTCGTGCAGGTCGCTACTTAT |
| *orf138* | ATTTGGCTAAGCTGGTTTTCT | CTCGGTCCATTTTCCACCTC |
| *orf224* | ATGCAATGATGGAGATGGAGT | CGATCAAGGTGCCGAAAGAT |

^a^ primers were cited from Kang et al. (2014)
