# Supplementary material for: A Novel Cytoplasmic Male Sterility in Brassica napus (inap CMS) with Carpelloid Stamens via Protoplast Fusion with Chinese Woad
Source: Front Plant Sci. 2017 Apr 6;8:529. doi: 10.3389/fpls.2017.00529 (PMC5382163; doi:10.3389/fpls.2017.00529)
Supplement: Supplementary file 2 [file Image_1.PDF]

Supplementary Material

A novel cytoplasmic male sterility in *Brassica napus* (inap CMS) with carpelloid stamens caused by mitochondrial DNA rearrangement via protoplast fusion with Chinese woad

Lei Kang *, Pengfei Li, Aifan Wang, Xianhong Ge, Zaiyun Li

*** Correspondence:** Zaiyun Li, lizaiyun@mail.hzau.edu.cn

1 Supplementary Figure

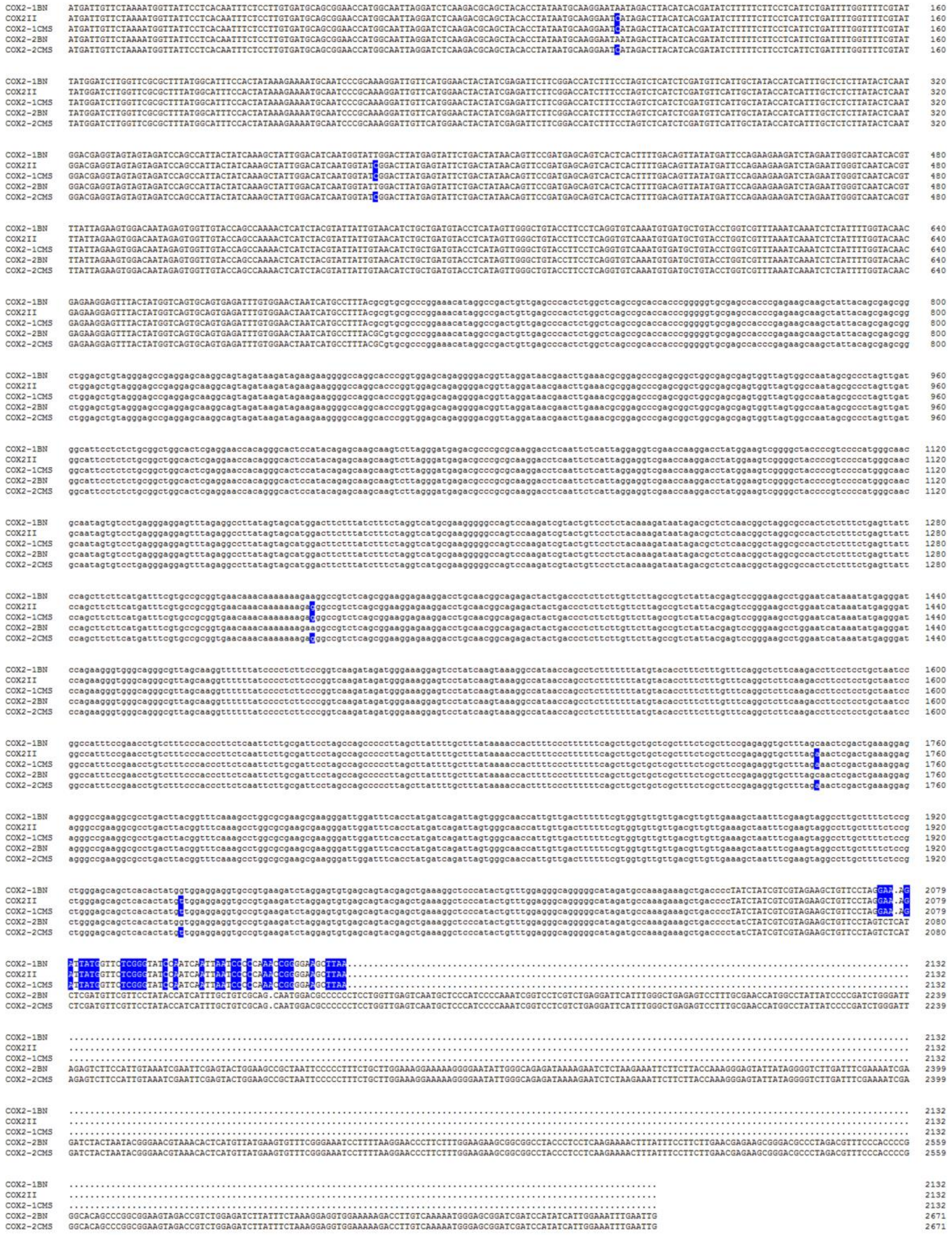

Supplementary Figure 1. Multiple sequence alignment of *cox2* copies in *B. napus*, *I. indigotica* and inap CMS. BN, *B. napus*. II, *I. indigotica*. CMS, inap CMS. Uppercase letters indicate the exon sequences. Lowercase letters indicate the intron sequences.
