# Supplementary material for: A Novel Cytoplasmic Male Sterility in Brassica napus (inap CMS) with Carpelloid Stamens via Protoplast Fusion with Chinese Woad
Source: Front Plant Sci. 2017 Apr 6;8:529. doi: 10.3389/fpls.2017.00529 (PMC5382163; doi:10.3389/fpls.2017.00529)
Supplement: Supplementary file 3 [file Image_2.PDF]

frontiers

Supplementary Material

A novel cytoplasmic male sterility in *Brassica napus* (inap CMS) with carpelloid stamens caused by mitochondrial DNA rearrangement via protoplast fusion with Chinese woad

Lei Kang *, Pengfei Li, Aifan Wang, Xianhong Ge, Zaiyun Li

*** Correspondence:** Zaiyun Li, lizaiyun@mail.hzau.edu.cn

1 Supplementary Figure

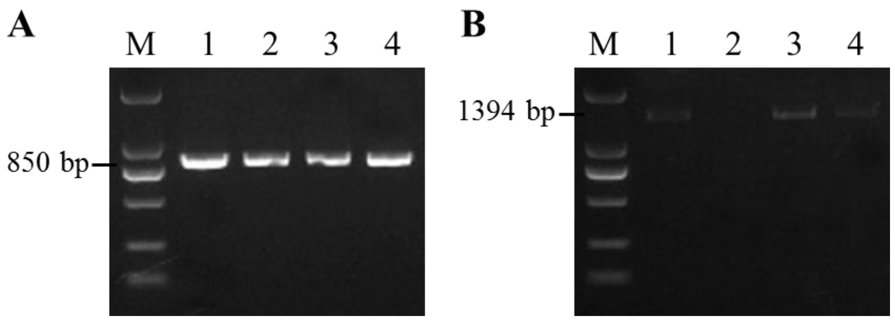

Supplemental Figure 2. RT-PCR analysis the expression of *cox2-1* and *cox2-2* in flower buds. **(A)** PCR amplification of *cox2-1* cDNA fragments using the primers F1/R1. **(B)** PCR amplification of *cox2-2* cDNA fragments using the primers F1/R2. M, DNA marker. 1, *B. napus*. 2, *I. indigotica*. 3, inap CMS. 4, addition line Me.
